# Impact of Wound Closure on the Corrosion Rate of Biodegradable Mg-Ca-Zn Alloys in the Oral Environment

**DOI:** 10.3390/ma13194226

**Published:** 2020-09-23

**Authors:** Ali Modabber, Daniela Zander, Naemi Zumdick, Daniel Schick, Kristian Kniha, Stephan Christian Möhlhenrich, Frank Hölzle, Evgeny Goloborodko

**Affiliations:** 1 Department of Oral, Maxillofacial and Facial Plastic Surgery, RWTH Aachen University Hospital, Pauwelsstr. 30, 52074 Aachen, Germany; kkniha@ukaachen.de (K.K.); fhoelzle@ukaachen.de (F.H.); egoloborodko@ukaachen.de (E.G.); 2 Chair of Corrosion and Corrosion Protection, Foundry Institute Aachen, Division of Materials Science and Engineering, RWTH Aachen University, Intzestr. 5, 52072 Aachen, Germany; d.zander@gi.rwth-aachen.de (D.Z.); n.zumdick@gi.rwth-aachen.de (N.Z.); 3 Department of Intensive Care Medicine, Medical Faculty, RWTH Aachen University, Pauwelsstr. 30, 52074 Aachen, Germany; dschick@ukaachen.de; 4 Department of Orthodontics, University of Witten/Herdecke, Alfred-Herrhausen Str. 50, 58448 Witten, Germany; Stephan.Moehlhenrich@uni-wh.de

**Keywords:** corrosion, Mg-Ca-Zn alloys, artificial saliva, wound closure, biodegradable implants

## Abstract

Magnesium alloys have exhibited a rapid rate of corrosion and thus early implant failure, so this study was designed to investigate the longer-term effects and in particular on wound closure. The aim of the study is to evaluate Mg-Ca-Zn Alloys as promising biodegradable implants in the field of maxillofacial surgery, which have so far never been evaluated for the changing conditions from a saliva to a serum-like environment after wound closure. Magnesium-0.6/calcium-0.8 wt.% zinc alloys were either immersed for 10 days in artificial saliva or 10 days in Hank’s salt solution as control groups. The test group was transferred from artificial saliva to Hank’s salt solution after 5 days in order to simulate wound closure. Corrosion rates were determined by immersion testing. Additional electron microscopy and energy dispersive X-ray spectroscopy (EDX) were performed. Prior artificial saliva exposure led to significantly decreased (*p* = 0.0272) corrosion rates after transfer to Hank’s solution in comparison to sole Hank’s solution exposure (0.1703 vs. 0.6675 mg/(cm^2^·day)) and sole artificial saliva exposure (0.3180 mg/(cm^2^·day)), which both exhibit a strong increase after 5 days. The results were in accordance with the scanning electron microscopy and EDX pictures. Prior saliva exposure could protect from increasing corrosion rates after wound closure. Thus Mg-Ca-Zn Alloys are promising future implant alloys in oral surgery, whereas other surgical fields without saliva exposure have to deal with accelerated corrosion rates after 5 days.

## 1. Introduction

Titanium and its alloys are commonly used as standard osteosynthetic materials in the field of maxillofacial surgery, but require secondary surgery in the case of metal removal [1]. Today development of new biodegradable metal implants is a highly investigated field in the medical research field, since resorption of implants would make a second intervention redundant. The first use of magnesium as an orthopedic biomaterial occurred at the beginning of the 20th century [2]. Unfortunately, those magnesium alloys showed a rapid corrosion rate and therefore early implant failure. Ever since, there have been many efforts to create special magnesium alloys to decrease this rate [2]. WE43 alloys possess mechanical properties similar to those of human bone and good corrosion results, but its elements yttrium and neodymium are still unexplored and require further research [3,4]. AZ91D as one example that already shows good corrosion resistance. It is said to show corrosion rates resulting in magnesium intake 43 times lower than the current human daily allowance, but on the other hand it is known to cause neurotoxicity [5,6].

Magnesium is a promising biodegradable metal for new implants, since its mechanical and physical properties resemble the human bone concerning its density, stability and modulus of elasticity [2,7,8]. Therefore, it is less likely to lead to stress shielding effects, which are observed in currently used alloys [7,9]. Earlier studies have already investigated the good mechanical and corrosion properties of magnesium-calcium-zinc (Mg-Ca-Zn) alloys [4,9,10,11,12], plus its components are naturally occurring elements, which all have important metabolic functions [13,14,15].

Former reports described beneficial effects to bone strength, growth, and callus forming without inflammatory side effects near the implant, which supports fracture healing [2,7,16,17]. The daily intake of magnesium in adults is around 300–400 mg and surplus intake is harmless and can be easily eliminated via the urine [9,15,18]. These aspects and the lack of need for secondary interventions through resorption make it the ideal implant for future maxillofacial surgeries.

However, positive effects are limited by the physiological blood or plasma environment, which tends to hydrogen evolution and alkalization through rapid corrosion due to high chloride amounts [19,20]. This may lead to delayed healing and necrosis of the tissue through hydrogen bubble formation and local alkalization [21,22]. Other studies show no adverse bubble forming effects [17]. Nevertheless, in order to solve these issues, the decrease of the corrosion rate, e.g., by special alloys, seems to be a promising step [21]. Witte et al. could show that hydrogen bubbles might be eliminated through the skin or dissolved in the blood in slow corrosion rates [23]. So far, many alloys or pure magnesium systems show relatively high corrosion rates, which limits their clinical application.

Implants in oral and maxillofacial surgery have to deal with different environmental properties through saliva exposure, which contains less chloride amounts than blood serum and is therefore most likely to resemble lower corrosion rates than reported in the literature for relevant chloride- containing electrolytes in medicine [6,10,19,24].

Studies investigating this aspect finished the studies after 5 days without investigating longer lasting effects and wound closure [10]. Physiologically this occurs on day 4–14 and leads to serum-like environmental conditions and therefore altered corrosion rates [25]. This study investigates those changing conditions after wound closure, by using a Hank’s salt solution, which simulates physiological pH and isotonic salt concentration. The impact of prior saliva exposure on the corrosion rate after wound closure is unknown and needs to be solved before application.

## 2. Materials and Methods

The protocol is in accordance with former experiments of our working group. The Mg-0.6Ca-0.8 wt.% Zn Alloys were developed from pure materials (99.94% Mg, 99.995% Zn and 99.8% Ca) [11]. Raw material was melted in a low-alloy steel crucible under argon and 2 vol.% SF_6_ with addition of calcium and zinc in the form of granules. The cast was heated to 750 °C for 10 min and then slagged off at 730 °C. The alloys were cast into a preheated mold at 720 °C of 380 mm × 35 mm × 22 mm. The exact composition of the alloys was measured via a spark spectrometer.

For the purpose of the immersion testing to obtain on one hand results on the hydrogen evolution rate and therefore on the directly correlated corrosion rate and on the other hand on the formation of corrosion layers dependent on the electrolytic conditions, test samples were cut wet to a 10 mm × 10 mm × 1 mm dimension using a corundum blade and were mounted on a non-conductible two-component-plastic based on methyl methacrylate. Afterwards the samples were ground with a SIC-Paper (up to 2400 grit), polished (water-free colloidal silica suspension) and mounted on an aluminum cylinder.

The immersion tests were maintained in artificial saliva based on the recipe of Klimek et al. [26] (Table 1). The solution was heated to 37 °C and constantly magnetically stirred. During the immersion experiment, the hydrogen evolution was monitored using a modified experimental setup based on a previous publication [27] by measure the developing H_2_ at specific time points in a burette.

During the corrosion process, the developing H_2_ was measured at specific time points in a burette. Assuming 1 mol of H_2_ corresponds to 1 mol of Mg the amount of developed H_2_ can be easily read out and gives direct information on the related corrosion rates. The pH-value of artificial saliva was kept constantly between 6.8 and 7.6 by adding phosphoric acid and potassium hydroxide. Corrosion rates were calculated by returning the slope of the linear regression line through the data in the specific time frame [10].

Mg-0.6Ca-0.8 wt.% Zn Alloys were either immersed for 10 days in artificial saliva (AS) or 10 days in Hank’s salt solution (HS), serving both as control groups. After 5 days the electrolyte was renewed due to cloudy liquid changes. For the test group (AS/HS), samples were firstly immersed into artificial saliva. Five days later they were transferred into Hank’s solution for 5 more days, simulating wound closure since the alloy would no longer be in contact with artificial saliva, but rather serum. Each experiment was repeated 3 times. The content of Hank’s solution is also displayed in Table 1. Its pH-value was kept constantly between 7.0–7.5 by adding phosphoric acid and potassium hydroxide.

A Zeiss Ultra 55 scanning electron microscopy with Oxford EDX (Carl Zeiss AG, Oberkochen, Germany) was used for the characterization of the microstructure and corrosion mechanism after 10 days. Quantitative data are presented as average plus the standard deviation. Statistical analysis was performed via SAS 9.4 (PROC GLIMMIX, SAS 9.4, SAS Institute Inc., Cary, NC, USA). Kolmogorov-Smirnov was used to assess normality. Either student’s t-test or one-way analysis of variance was further used to assess significance. In order to check for multiple comparisons, Tukey’s method was used as a post hoc test. A *p*-value below <0.05 was determined as significant and is indicated by an asterisk. Further details are received below the figures.

## 3. Results

**Figure 1:**
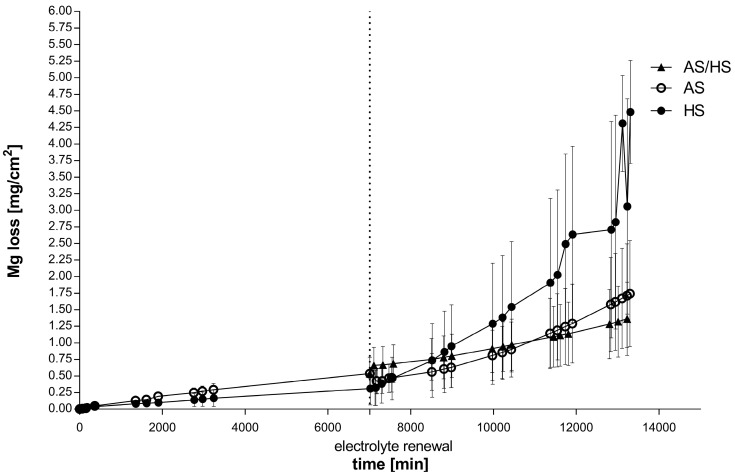
10-day-corrosion curves of Mg-0.6Ca-0.8Zn wt.% in Hank’s balanced solution, artificial saliva as well as AS/HS (5 days artificial saliva, 5 days Hank’s balanced solution) Electrolyte contents of the control groups were renewed after 5 days. Each experiment was repeated at least 3 times. Values are shown as means.

**Figure 2:**
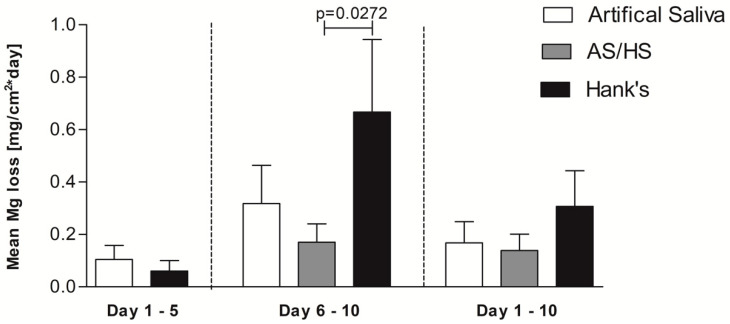
Displayed are the calculated corrosion rates as mean magnesium loss per day of Mg-0.6Ca-0.8Zn wt.% either for day 1–5, day 6–10 after the electrolyte change or for day 1–10. Each experiment was repeated at least 3 times. Values are shown as means plus standard deviation.

The corrosion kinetics in Figure 1 show the indirect Mg mass loss over the time by measurement of hydrogen evolution, whereas in Figure 2 and Table 2 the mean corrosion rates per day of the single electrolytes are displayed. Initially corrosion rates between artificial saliva and Hank’s solution exhibit a similar appearance for the first 5 days, as no significant difference concerning early corrosion rate could be determined. At the time of the electrolyte change both artificial saliva curves show a higher mass loss (0.53 mg/cm^2^) than the alloys in Hank’s solution (0.32 mg/cm^2^). After 5 days the mass loss in Hank’s solution increases strongly and outreaches the other alloys after approximately 6 days. Accordingly, the corrosion rate rises. This trend continues and at the end of the measurements Hank’s solution exhibits approximately more than double the magnesium mass loss of AS/HS (2.82 vs. 1.36 mg/cm^2^). In contrast to the steep corrosion increase in Hank’s solution, electrolyte change from artificial saliva to Hank’s solution does not seem to affect the corrosion rate of those immersed alloys, as they continue with almost the same rate. On the other hand, alloys which are placed permanently into artificial saliva show an increase after electrolyte change, with an even higher corrosion rate (0.3180 ± 0.1457 mg/(cm^2^·day)) than alloys, which were immersed in AS/HS (0.1703 ± 0.06967). Nevertheless, regarding the kinetics, both those two environmental conditions keep constantly below the curve of Hank’s solution.

Focusing on the rate after the fifth day, the corrosion rate in Hank’s solution exceeds the others with 0.6675 ± 0.2766 mg/(cm^2^·day) as observed previously in the kinetics. The difference between alloys, which were only immersed into Hank’s solution and alloys in AS/HS is significant (*p* = 0.0272), whereas the latter alloys show the lowest corrosion rate of all.

The mean corrosion rate from day 1–10 is displayed on the right side of Figure 2 and Table 2. Overall alloys immersed in only Hank’s solution exhibit the highest corrosion rate with 0.3073 ± 0.1356 mg/(cm^2^·day). Again, alloys in AS/HS show the lowest rate with 0.1385 ± 0.06277 mg/(cm^2^·day), although this is not significant.

**Figure 3:**
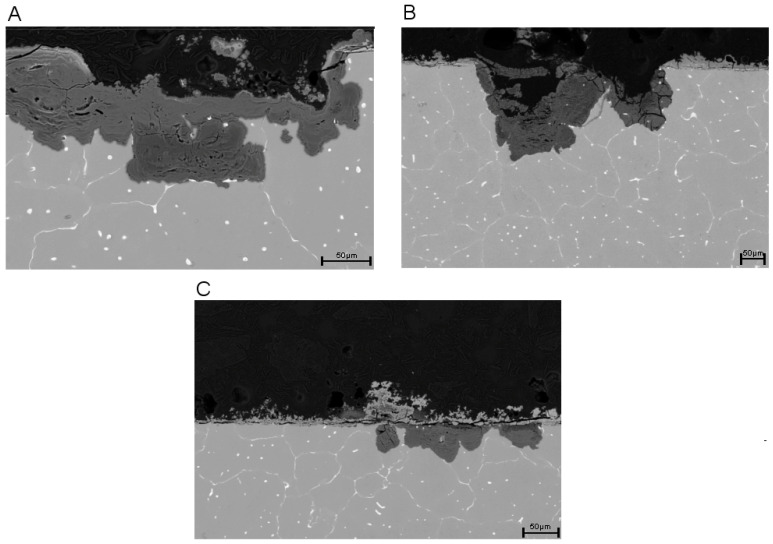
Scanning electron micrographs of the microstructure of Mg-0.6Ca-0.8 wt.% Zn Alloys for (**A**) 10 days in Hank’s solution, (**B**) 10 days in artificial saliva or (**C**) in AS/HS (5 days artificial saliva, 5 days Hank’s balanced solution).

**Figure 4:**
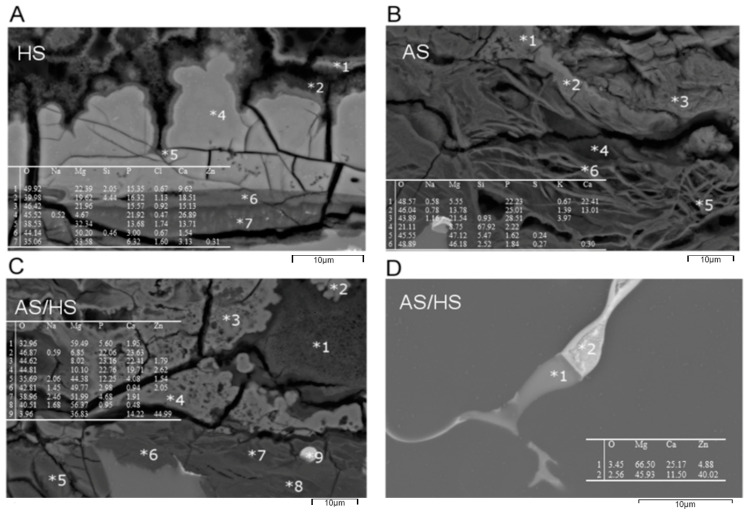
Cross-sectional scanning electron micrograph with EDX Analysis of Mg-0.6Ca-0.8 wt.% Zn Alloys for (**A**) 10 days in Hank’s solution, (**B**) 10 days in artificial saliva or (**C**,**D**) in AS/HS (5 days artificial saliva, 5 days Hank’s balanced solution).

Figure 3 shows SEM cross section pictures of the three differently immersed alloys after 10 days. The pictures are in accordance with the previously measured corrosion rates after 10 days (Figure 1). Alloys which were completely immersed into Hank’s solution for 10 days (A) clearly show a larger localized corrosion attack in comparison to the others. Therefore, also the formation of a higher volume of corrosion products within the corrosion pits which are formed by selective dissolution of phases was observed. In addition the formation of a corrosion layer was observed on top of the surface from own previous studies [10] it is well known that the corrosion mechanism is driven on one hand by the existing Mg_6_Ca_2_Zn_3_, α-Mg, Mg_2_Ca phases as well as by the formation of corrosion products. It was confirmed that the dissolution of the phases follows the electrochemical potentials according to the nobility: Mg_6_Ca_2_Zn_3_ > α-Mg > Mg_2_Ca. Therefore, the dissolution starts with Mg_2_Ca followed by Mg Figure 4D confirms the formation of the secondary binary and ternary phase within the Mg matrix. However, it also indicates that the binary Mg_2_Ca phase is enriched in Zn resulting in an increased nobility of this phase in the investigated alloy.

The samples which were immersed in artificial saliva revealed similar corrosion attacks in comparison to samples immersed in Hank’s solution. However, the distribution of localized corrosion attacks is lower (B) and reflect a slower degradation rate compared to the results in Hank’s solution (Figure 2).

The AS/HS group exhibits only a small amount of areas where selective corrosion takes place and the formation of a very homogenous distributed surface layer was observed. The latter one is assumed to affect the corrosion mechanism significantly. However, it is assumed that the corrosion layer formed during pre-conditioning in artificial saliva on top of the surface acts more protective than the corrosion layer formed in Hank’s solution. For the AS/HS samples an almost homogenous surface layer (C) is assumed to lead to a significantly reduction of the mass loss as well as to a slow and linear corrosion kinetics during the following immersion in Hank’s solution.

In Figure 4 cross-sectional scanning electron micrographs images in higher resolution with corresponding EDX analysis of specific corrosion product points are shown. Areas close to the surface (picture A–C; points 1–3) show lesser amounts of magnesium, whereas oxygen, phosphate and calcium contents within the corrosion products are elevated. The Ca/P ratio ranges from 2.0 till 1.0 and Mg/O is approximately 0.5 for (A) and (B), whereas (C) exhibits 0.2. Persistent through all three electrolytes is a magnesium gradient within the corrosion products, which increases continuously by approaching areas close to the not corroded Mg phase (points 4–8). Those points reveal mostly a Mg/O ratio of 1.0. On the other hand, phosphate and calcium contents decrease continuously when reaching the not corroded inner matrix.

Surface-related areas additionally show voluminous cracks in all three electrolytes. Summarizing the EDX results the corrosion products are assumed to consist on one hand of magnesium hydroxide or magnesium oxide in the areas where selective corrosion of the Mg-phase occurred and on the other hand on a calcium-magnesium-phosphate which is formed at the surface of the alloy. However, it also was observed that there is a strong variation within the calcium-magnesium-phosphate layer in regard to the magnesium content, indicating variations in the stoichiometry of the formed phosphate. In addition, this corrosion layer at the surface is considered to be to some extent permeable for chloride-ions which is indicated by the EDX measurements (A) in Hank’s solution.

## 4. Discussion

Former studies of Mg-0.6 wt.% Ca-0.8 wt.% Zn and Mg-0.6 wt.% Ca-1.8 wt.% Zn revealed the corrosion behavior in artificial saliva of different composition (±mucin, ±urea) [28]. Here a very low corrosion rate was found due to a lower Zn content of the investigated alloys and the influence of the artificial saliva chemistry. Specially, it was observed that the combination of mucin and urea served as corrosion inhibitors due to the formation of a thin, homogenous corrosion layer with protective qualities [29,30]. Maxillofacial implants require a slow and homogenous corrosion rate in order to prevent early implant failure and negative side effects. Therefore, within this study Mg-0.6 wt.% Ca-0.8 wt.% Zn was investigated by using hydrogen evolution measurements in Hank’s salt solution, artificial saliva solution and the combination of both by replacing the artificial saliva solution with the Hank’s salt solution after 5 days. Only the hydrogen evolution test was used to quantify the corrosion rate and to characterize the corrosion behavior because it is well known that e.g., DC testing for magnesium alloys reflect several disadvantages, such as results strongly deviating by as much as 50–90% [31] or corrosion current densities which occur often much lower in comparison to the immersion test due to overlaying kinetic effects.

The observed corrosion results revealed that in the first 5 days of the immersion experiment, the hydrogen evolution rate of the two different electrolytes kept constantly stable and nearly similar until the time of electrolyte change (Figure 2). Previously published immersion experiments with the same alloy in Hank’s balanced solution are in accordance with this result [10]. Nevertheless, our results for 5 day immersion experiments in artificial saliva differ from earlier trials, which proclaimed them to be lower than in Hank’s balanced solution, with a rate of 0.010 ± 0.004 mg/(cm^2^·day) and therefore in the range of the previously estimated tolerable corrosion rate suggested by Song et al. [21]. Yet our results indicate much higher corrosion rates and thus similar to our Hank’s solution corrosion rate. Our data were collected from three simultaneously performed experiments. Therefore, we expect them to be more accurate, since previously published results were determined in separately performed experiments.

Nevertheless, Hank’s solution was predicted frequently in earlier literature to exhibit a higher corrosion attack and rate. This was related to the observations in chloride containing electrolytes, either in Hank’s solution or in NaCl electrolytes. In general it was estimated that a higher chloride concentration, which is also relevant in Hank’s solution (>30 mmol/L), is able to dissolve, e.g., Mg(OH)_2_, corrosion products/layers and therefore lead to further corrosion enhancement by the breakdown of protective layers [2,6,10,19,32,33]. The effect of breakdown of the observed calcium-magnesium-phosphate layer due to chloride penetration is assumed to be associated to the strongly, almost exponential, accelerated mass loss of the investigated Mg-Ca-Zn alloy at the time of the electrolyte change, when the corrosion rate in Hank’s solution significantly exceeds the rate in AS/HS (Figure 2). This was confirmed by the scanning electron microscopy (Figure 3A), which show the formation of additional Mg(OH)_2_ or MgO corrosion products underneath the phosphate layer due to microstructural dependent selective corrosion mechanisms. Interestingly, previous artificial saliva immersion seems to have a protective effect on the alloy. Thus, transposition from artificial saliva into Hank’s balanced solutions leads to the lowest corrosion rate of all three electrolytes (0.1703 ± 0.06967) and is therefore significantly lower than in alloys, which were exposed to Hank’s solution only. This is also in accordance with the scanning electron microscopy results, which show less selective corrosion attack and far more uniform corrosion for this electrolyte (Figure 3C) in comparison to sole Hank’s solution exposure (Figure 3A). Therefore, especially strong localized corrosion as well as the breakdown of the corrosion layer on the surface leads to rate accelerations as seen especially in Figure 3A for sole Hank’s balanced solution exposure.

However, this study indicates that there seems to be a general increase in corrosion rates for all three tested solutions after 5 days, even though less exceedingly as in sole Hank’s solution exposure. Whether this effect is due to the electrolyte change or a general phenomenon needs to be further investigated, as the electrolyte change is unavoidable due to cloudy liquid changes and thus inadequate measurement conditions. Also, at this point it is unclear, whether artificial saliva’s protective qualities are long lasting. This needs to be further investigated in longer lasting trials, since exposure in Hank’s solutions only accelerated after 5 days and Mg-0.6 wt.% Ca-0.8 wt.% Zn previously immersed in artificial saliva were held in Hank’s solution afterwards for 5 more days.

However, the influence of the microstructure on the selective corrosion mechanism as well as the formation of a calcium-magnesium-phosphate layer on our results follows previous publications [10,34,35]. Figure 4D proves the existence of the secondary phases, Mg_2_Ca partially enriched with zinc (point 1) and Mg_6_Ca_2_Zn_3_ (point 2). Since the previously claimed hypothesis in which electrochemical potentials follow Mg_6_Ca_2_Zn_3_ > α-Mg > Mg_2_Ca [10] can be confirmed, it is assumed that Mg_2_Ca enriched with Zn as well as the Mg phase is dissolved in the first stage after immersion and contributes to the observed dissolution rates.. However, it is assumed that directly after immersion already a first calcium-magnesium-phosphate surface layer is formed and growth with time on Mg-0.6 wt.% Ca-0.8 wt.% Zn dependent on the chosen electrolyte composition. In general variations of the calcium and magnesium concentration from the surface near region towards the inner microstructure was observed. Those could indicate the formation of different calcium phosphate salts e.g., CaHPO_4_(2H_2_O), octacalcium phosphate (OCP), tricalcium phosphate (TCP) and hydroxyapatite (HA), which stand for progressive corrosion stages. However, important is also to note that the calcium phosphates which were formed, consist of magnesium with various concentrations indicating different stoichiometries. This variation in stoichiometry is assumed to influence the ability of chloride to penetrate towards the inner interface between phosphate and metal. In addition, areas close to the metal interface show decreased calcium and phosphate contents, while being mainly consistent of magnesium and oxygen. This could indicate either rather early stage selective corrosion or continuous selective corrosion during the whole immersion time by penetration of the chloride containing electrolyte components through the calcium-magnesium-posphate layer. Both should results in the formation of Mg(OH)_2_ or MgO as corrosion products in the vicinity of the former Mg phase.. This gradient is persistent throughout all three electrolytes. Additionally, all pictures show surface related voluminous cracks, which could be either the result of drying or distinctive hydrogen evolution [10,35]. Since only Mg-0.6Ca-0.8 wt.% Zn Alloys were used for all three different electrolytes and no large difference could be observed concerning their microstructural dependent type of selective corrosion mechanism but a large difference regarding the intensity of the selective corrosion phenomena, we state that the significant changes in the corrosion resistivity can be associated to the local structural and stoichiometric differences of the observed calcium-magnesium-phosphate layer which is formed under the different electrolytic conditions. This is assumed to be directly related to a specific defect structure, e.g., associated to voids at a smaller scale and cracks at a bigger scale, which forms differently with an increasing amount of Na_2_HPO_4_ and KH_2_PO_4_, a decreasing amount of chloride ions and the addition of mucine and urine. The different rates therefore have to be the result of locally occurring corrosion acceleration driven by the local chloride concentration, the differences in the structure and chemistry of the phosphate layers and the breakdown of those layers.

In addition it has to be mentioned, that the immersion experiments were performed for 10 days. Further research needs to be done to investigate the exact mechanism behind the upper described phenomena and corrosion rate behavior in longer trials. Also, the general rate has to be decreased to reach the desired rate by Song et al. [27]. So far efforts undertaken to tailor corrosion rate already show decreased hydrogen evolution in vitro and in vivo. Those include avoidance of trace impurities, increasing solidification cooling rates and heat treatment [32,36,37,38]. For instance trace impurities are incorporated into intermetallic phases and lead to additional cathodic reactivity especially in the initial period [39]. Increasing solidification cooling rate also increases corrosion resistance by decreasing average grain size, developing more dispersive and continuous secondary phases, homogenizing corrosion mechanism and enhancing microstructural refinement. Homogenization of the microstructure can also be achieved by heat treatment.

After wound closure and thus serum-like environmental conditions preincubation in artificial saliva could protect from increasing corrosion rates, which had been observed especially in sole Hank’s solution and thus facilitates uniform corrosion. The slow and uniform corrosion protects from early implant failure, because especially the localized corrosion seem to corrode quickly. Therefore, fewer negative side effects through bubble forming, alkalization or local necrosis occur.

## 5. Conclusions

Those properties emphasize the future use of Mg-0.6Ca-0.8 wt.% Zn Alloys especially in maxillofacial surgery in comparison to other applications, where a sole serum-like environment (e.g., orthopedic field) may contribute to higher corrosion rates after 5 days. Patients could benefit from its absorbable material and would not have to undergo secondary plate removal surgery.

## Figures and Tables

**Table 1:** Content of artificial saliva and Hank’s solution in mg/L.

	NaCl	Na_2_HPO_4_	KH_2_PO_4_	CaCl_2_	Glucose	Phenol Red	NaHCO_3_	KCl	MgSO_4_ · 7H_2_O	
**Hank’s solution**	8000	38	60	140	1000	10	350	400	200	
	**NaCl**	**Na_2_HPO_4_**	**KH_2_PO_4_**	**CaCl_2_**	**Glucose**	**NaSCN**	**NH_4_Cl**	**Asorbic**	**Urea**	**Mucin**
**Artificial Saliva**	580	340	330	128	30	160	160	2	200	2700

**Table 2:** Shown are the calculated corrosion rates as mean Magnesium loss per day of Mg-0.6Ca-0.8Zn wt.% with standard deviation either from day 1–5, after the electrolyte change (day 6–10) or from day 1–10.

	Day 1–5		Day 6–10			Day 1–10		
Medium	AS	HS	AS	AS/HS	HS	AS	AS/HS	HS
**Mean**·(mg/(cm^2^ × day)) **± SD**	0.11 ± 0.05	0.06 ± 0.04	0.32 ± 0.15	0.17 ± 0.07	0.67 ± 0.28	0.17 ± 0.08	0.14 ± 0.06	0.30 ± 0.14
